# Mixing and Aeration Effects in Outdoor Dual-Chamber Microbial Fuel Cells with Agarose Salt Bridges

**DOI:** 10.3390/membranes16080275

**Published:** 2026-08-18

**Authors:** Mohamad K. Khawaja, Nour Alnajjar, Ammar Alkhalidi

**Affiliations:** 1 Electrical and Energy Engineering Department, German Jordanian University, Amman 11180, Jordan; 2 Institute for Power Electronics and Electrical Drives (ISEA), RWTH Aachen University, 52074 Aachen, Germany; 3 Sustainable & Renewable Energy Engineering Department, University of Sharjah, Sharjah 27272, United Arab Emirates; 4 Sustainable Energy & Power Systems Research Centre, RISE, University of Sharjah, Sharjah 27272, United Arab Emirates; 5 Mechanical and Maintenance Engineering Department, German Jordanian University, Amman 11180, Jordan

**Keywords:** aeration, agarose salt-bridge separator, microbial fuel cells, mixing, power generation, wastewater treatment

## Abstract

Microbial fuel cells (MFCs) provide a membrane-based bioelectrochemical route for simultaneous wastewater treatment and electricity generation. This study investigates the effect of aeration and mixing on the performance of dual-chamber microbial fuel cells (DCMFCs) operated outdoors using sugar-mix substrates. Five DCMFC configurations were evaluated over 40 days, including baseline operation and individual or combined aeration and mixing strategies. Voltage and current were recorded every 15 min, while solar insolation and ambient temperature were monitored to assess environmental effects. Chemical oxygen demand (COD) was measured to evaluate wastewater treatment performance. The configuration with continuous aeration and mixing achieved the best performance, reaching a maximum voltage of 563.2 mV and a peak power output of 250.58 µW. Compared with baseline Cell 1, Cell 5 showed a 72.5% higher Week 6 maximum power density. The final COD concentration in Cell 5 was 12.4% lower than that measured in baseline Cell 1; this represents an endpoint difference rather than a reactor-specific COD removal efficiency. Exploratory correlation analysis showed configuration-dependent associations between electrical output and ambient conditions but did not identify a consistent positive relationship between solar insolation and power generation. These results demonstrate that combined aeration and mixing can improve DCMFC performance under realistic outdoor conditions and support the development of scalable, low-resource systems for decentralized bioenergy generation and wastewater treatment.

## 1. Introduction

Fossil fuel proved to be a profound catalyst in global development, but it came with substantial environmental repercussions. As of 2019, carbon dioxide emissions from fossil fuel combustion soared to 36.7 billion metric tons, highlighting an upward trajectory characteristic of the 21st century [1]. Concurrently, the looming finitude of fossil fuel reserves accentuates the urgency for sustainable alternatives. Within the context of renewable energy discussions and emerging technologies, fuel cell technology has emerged as an area with substantial academic interest and practical field applications. Fuel cells (FCs) are devices that convert chemical energy into electrical energy through electrochemical reactions. They operate by utilizing a fuel, typically hydrogen, and an oxidizing agent, usually oxygen, to produce electricity, water, and heat as byproducts. The basic principle of operation involves the fuel being oxidized at the anode, releasing electrons that travel through an external circuit to the cathode, where they combine with the oxidizing agent. FCs, distinguished by their electricity generation through electrochemical reactions involving hydrogen and an oxidant, continuously generate power if the requisite fuel and oxidant are supplied, sidestepping the Carnot efficiency limitation, essential to combustion-based systems, which restricts the maximum possible efficiency of these systems due to heat losses and the practical constraints.

Fuel cell research has developed from early electrochemical systems into a range of technologies for stationary, transport, and specialized applications. The spectrum of fuel cell technology, enriched by diverse types such as proton exchange membrane fuel cells (PEMFCs), solid oxide fuel cells (SOFCs), alkaline fuel cells (AFCs), and molten carbonate fuel cells (MCFCs), showcases the expansive potential of fuel cells [2].

In the scope of fuel cell taxonomy, microbial fuel cells (MFCs), a distinct subgroup of bio-electrochemical systems (BESs), employ bacteria as catalysts to transform chemical energy into electrical energy. Mainly, MFCs utilize organic waste like low-strength wastewater and lignocellulosic biomass for electricity generation, harnessing electrons from the anaerobic oxidation of substrates driven by bacterial-catalyzed biochemical reactions [3]. MFC performance is governed by interacting biological, electrochemical, and design factors, including microbial community composition, substrate characteristics, electrode and separator properties, temperature, pH, and external resistance [4,5]. MFCs may employ selected electroactive cultures or mixed microbial consortia obtained from wastewater, sludge, sediment, or animal waste. Recent work has confirmed that cow dung can serve both as an organic substrate and as a source of microorganisms for bioelectricity generation in MFCs [6]. In the present study, the cow manure provided an indigenous mixed microbial inoculum; individual microbial species were not isolated or taxonomically characterized.

Substantial improvements have been made to highlight the simplicity, adaptability, and potential of MFCs in power generation and waste management. The adoption of graphene, for instance, has been shown to enhance system output parameters significantly [7]. Concurrently, a body of research has explored crucial aspects, including electrode arrangement, organic substrate supply, construction material selection, and the application of various substrates, such as cow dung and molasses, in MFCs, each revealing potential for enhancing power density and remediation efficiency [8,9]. Aeration and substrate mixing in MFCs have shown potential in improving power generation and contaminant removal but their combined operational influence in outdoor, real-world settings remains insufficiently examined.

Recent experimental studies confirm that cathodic aeration and anodic hydrodynamics can influence oxygen availability, mass transport, biomass suspension, substrate distribution, and electrical performance. However, the resulting effects depend on the aeration regime, mixing intensity, external resistance, substrate characteristics, and reactor configuration [10,11]. Microbial Fuel Cells (MFCs), a class of bio-electrochemical systems, have drawn attention for their ability to generate electricity from organic matter while simultaneously treating wastewater [3]. Unlike traditional fuel cells, MFCs utilize microorganisms to drive electrochemical reactions. Various configurations have been investigated, including single-chamber, dual-chamber, and stacked designs [12,13,14]. Research has also examined the effects of different substrates [15], electrode and membrane materials [16], and operational parameters such as temperature and external resistance [17]. Additional enhancements such as aeration in the cathodic chamber [3,18], substrate mixing in the anodic chamber [19], and incorporation of advanced electrode materials and catalysts [7] have demonstrated potential to boost both power output and treatment efficiency.

The separator is a central component of a dual-chamber MFC because it enables ionic transport while maintaining distinct anodic and cathodic reaction environments. Recent membrane research identifies ionic conductivity, ion selectivity, membrane resistance, oxygen and substrate crossover, biofouling, mechanical stability, durability, and material cost as important considerations in MFC separator selection [20]. Long-term experimental evidence further demonstrates that separator properties and stability can influence internal resistance, organic-matter retention, and sustained electrical and treatment performance [21]. In the present study, the agarose–KCl–NaCl salt bridge was prepared using the same composition, geometry, and setting procedure for all five cells. It was therefore maintained as a fixed reactor component rather than investigated as an independent experimental variable. Beyond bioelectrochemical systems, recent wastewater-remediation research has explored multifunctional treatment platforms capable of combining contaminant degradation with microbial disinfection. These include solar-driven photothermal catalytic treatment and contact-electrocatalytic systems for the simultaneous removal of microorganisms and micropollutants [22,23]. Although these technologies operate through mechanisms different from MFCs, they illustrate the broader development of integrated treatment systems targeting both chemical and biological contamination.

Previous studies have examined cathodic aeration, anodic mixing, substrate composition, and MFC construction as individual design or operating factors. However, comparatively fewer studies have evaluated these strategies side by side using the same reactor design, substrate, separator, and monitoring approach under uncontrolled outdoor conditions.

This study compares five DCMFC operating configurations over a 40-day outdoor period: passive operation, controlled cathodic aeration, controlled anodic mixing, controlled combined operation, and continuous combined operation. All reactors used the same cow-manure–beetroot-molasses substrate and agarose salt-bridge separator. The contribution of the study lies in the comparative assessment of electrical performance and final COD behaviour under equivalent outdoor operating conditions. The study is intended to provide application-oriented evidence relevant to field operation and future system refinement rather than to propose a new electrochemical mechanism.

## 2. Methodology

This study utilized an experimental design to evaluate the bioelectricity generation potential of dual-chamber microbial fuel cells (DCMFCs). The DCMFC model comprised an anaerobic anode chamber and an aerated cathode chamber, separated by a proton exchange membrane (PEM). Each DCMFC unit was assembled in a stepwise process, beginning with chamber assembly, PEM casting, and electrode installation, followed by mixer and aerator setup based on configuration. In the anode chamber, microorganisms from the manure convert chemicals into bioelectricity through anaerobic respiration, producing electrons and protons. Electrons navigate through an external circuit linked to the cathode surface and facilitated by the PEM, uniting with protons and reacting with dissolved oxygen to form water.

Five DCMFC designs were tested to examine how different operational components affect performance. Each cell featured a different configuration and schedule for two active elements: a mixer in the anode chamber and an aerator in the cathode chamber.

- **Cell 1** served as the control, with no active mixing or aeration.
- **Cell 2** included only an aerator in the cathode chamber.
- **Cell 3** used only a mixer in the anode chamber.
- **Cell 4** combined both a mixer and an aerator, but with controlled (non-continuous) operation.
- **Cell 5** also included both components, operating continuously for 24 h.

**Figure 1:**
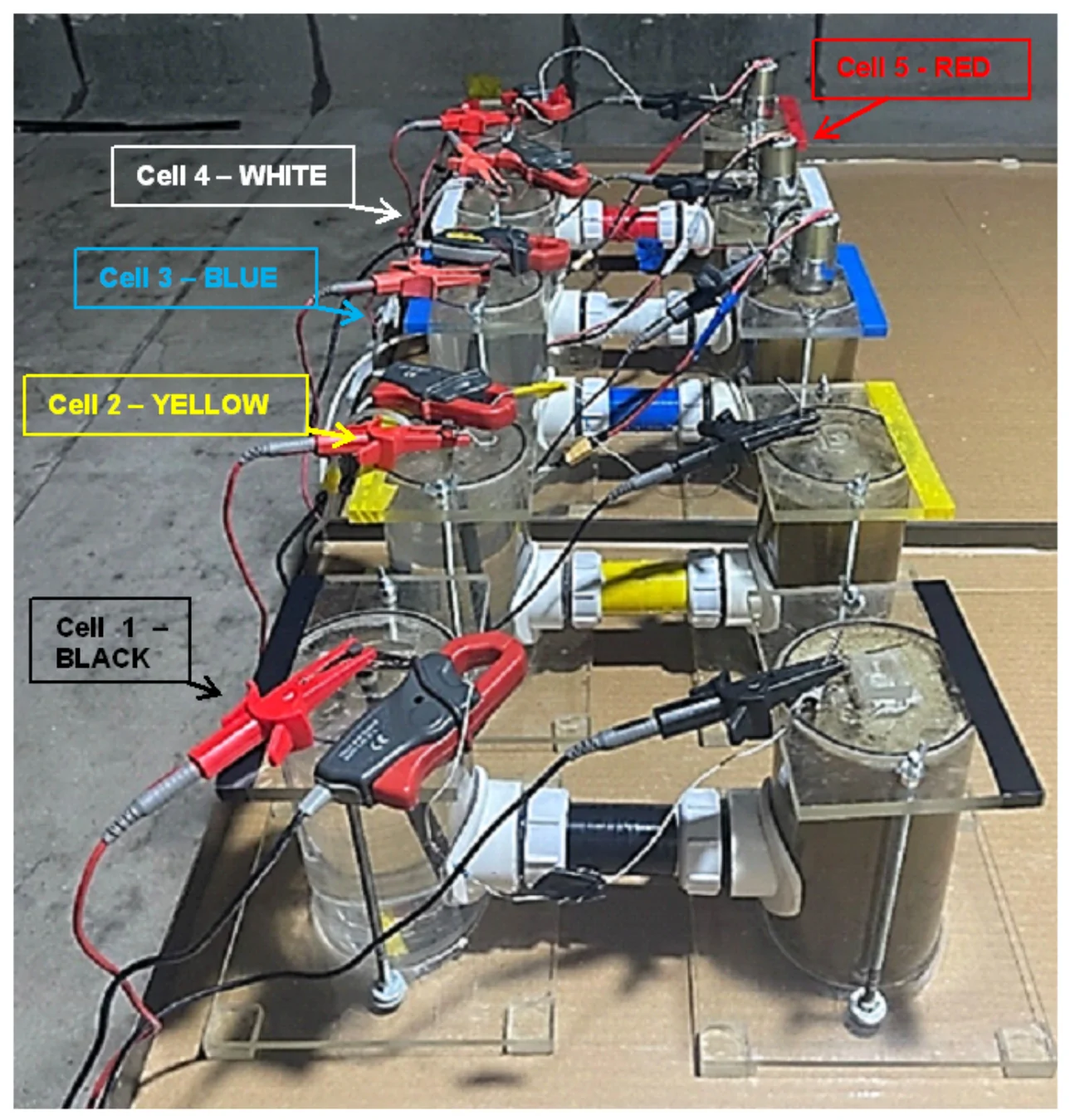
Experimental setup.

The aerator introduced oxygen into the cathode chamber, a critical element for the final step of electricity generation, where electrons, protons, and oxygen combine to form water. Greater oxygen availability may enhance this reaction and increase electrical output. Meanwhile, the mixer in the anode chamber helped maintain a uniform distribution of microorganisms and organic substrate, supporting consistent biological activity and efficient breakdown of organic matter. By testing these variations, the study enabled a comparative assessment of the individual and combined effects of aeration and mixing on MFC performance under real-world conditions. Figure 1 shows the overall setup of the experiment, and Table 1 highlights the different cell configurations.

Table 1 summarizes the active components and daily operating schedule applied to each DCMFC configuration. The aerator facilitated the introduction of oxygen, crucial for the final step of electricity generation, where electrons combine with protons and oxygen to form water. This enhanced oxygen may increase the potential for electricity generation. On the other hand, the mixer ensures a uniform distribution of microorganisms and substrate within the anode chamber, promoting efficient organic matter breakdown and possibly consistent and increased electricity output. This variation allowed comparative testing of individual and combined operational components (aeration and mixing) on MFC performance.

The study spanned from 24 September to 2 November 2022. All cells were positioned side by side and exposed to the same general outdoor environment; however, substrate temperatures within the individual anode and cathode chambers were not monitored or actively controlled. Two strategies were adopted: 24-h continuous aeration and mixing and controlled 5-h daytime operation to harness peak daytime temperatures for maximal efficiency [3,18,24,25]. A digital multimeter (CEM DT-965) captured voltage and current measurements at 15-min intervals from 7 AM to 4 PM. The solar intensity, temperature, and wind speed were logged three times daily (7:30 AM, 12 PM, and 3:30 PM). One final chemical oxygen demand (COD) sample was collected from each reactor after 40 days. No intermediate COD samples were collected, and the final concentrations were compared descriptively among the five configurations. No water, substrate, or replacement liquid was added during the experimental period. An Excel-based tracking sheet was developed and used throughout the experiment to log procedures and sampling schedules in real-time, ensuring a timely, structured, and traceable execution of all experimental steps. The experimental procedure was repeated on three occasions under comparable conditions as a reproducibility check. The repeat tests showed similar electrical-performance trends, with approximately 2% variation in maximum power. These tests were conducted sequentially rather than as parallel biological reactor replicates. The results presented are from the principal 40-day experimental campaign and are not averages of the three repeat tests. In the principal campaign, one reactor represented each operating configuration. Consequently, comparisons among configurations are presented as descriptive observations under the tested conditions rather than as statistically confirmed treatment effects. The collected data were analysed using Microsoft Excel and Power BI for descriptive analysis and graphical presentation. Pearson correlation coefficients and two-sided p-values were calculated between daily maximum power and the corresponding noon solar-insolation and ambient-temperature measurements across the 40-day experimental period. These correlations were treated as exploratory because environmental conditions and reactor development changed concurrently over time.

Instantaneous power under the fixed 1 kΩ external load was calculated from the measured voltage and current. With voltage expressed in millivolts and current in microamperes, power was calculated using Equation (1). Power density was calculated by dividing power by the manufacturer-specified carbon-brush surface area of 1.06 m^2^ and is reported in µW/m^2^, as shown in Equation (2). Current density was calculated using the same normalization area and is reported in µW/m^2^, as shown in Equation (3). The external resistance was not varied; therefore, the reported power values represent output under the fixed 1 kΩ load rather than confirmed maximum-power-point values.

(1) P=V×I,

(2) Pd=P/A,

(3) J=I/A,

### 2.1. DCMFC Setup

The anode and cathode chambers were constructed from plexiglass cylinders, each with a volume of 1500 mL and measuring 19 cm in height and 10 cm in diameter. These cylinders were sealed using a square-shaped lid and a watertight inner fabricated small cylinder that fits into the larger one, incorporated with a rubber band and two scroll rods with bolts for improved sealing. To facilitate proton exchange, a 12 cm PVC pipe of 3.5 cm diameter was attached between the two chambers. Secure connections were ensured using silicone sealants and male-female couplings. To accommodate the aerator and mixer, the top lid of the respective compartments had a hole for the aerator sized per the aerator hose and a 6 mm sealed opening for the mixer rod. A specifically designed plexiglass base with a groove guaranteed the mixer’s alignment during rotation.

### 2.2. PEM Preparation

**Figure 2:**
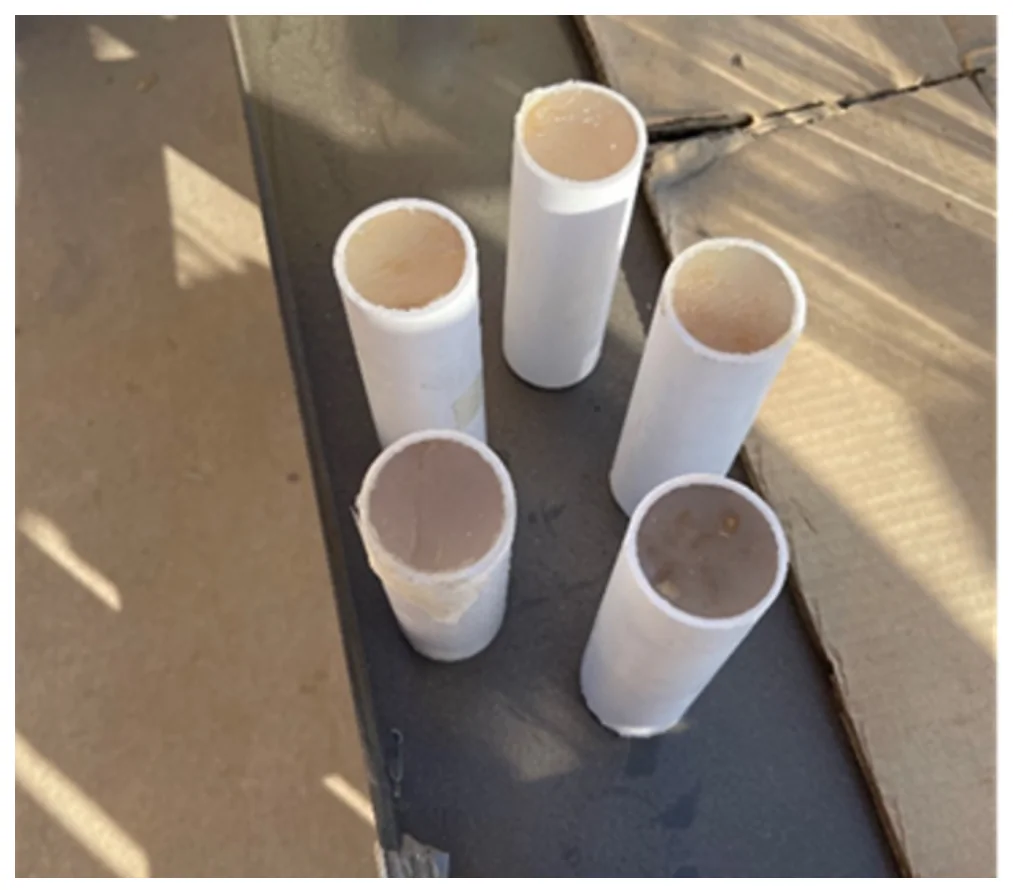
Preparation of the agarose–KCl–NaCl salt-bridge separator before installation between the anode and cathode chambers.

The ion-conducting separator was prepared as a 10% agarose salt bridge. Powdered agarose was added to heated water under continuous mixing, followed by 1 mol of potassium chloride and 1 mol of sodium chloride [26,27,28,29]. While still hot, the mixture was poured into a PVC tube measuring 12 cm in length and 3.5 cm in diameter and was allowed to solidify for 24 h. The salt bridge enabled ionic transfer between the two chambers while maintaining their physical separation. The same composition, dimensions, preparation method, and setting period were applied to all five cells. The separator was therefore maintained as a fixed reactor component rather than varied as an experimental factor. The tube was then secured between the anode and cathode chambers using male–female couplings and silicone sealant. The solidified agarose in the PVC then acted as a salt bridge, facilitating proton transfer as shown in Figure 2.

### 2.3. Substrate and Electrode Preparation

**Figure 3:**
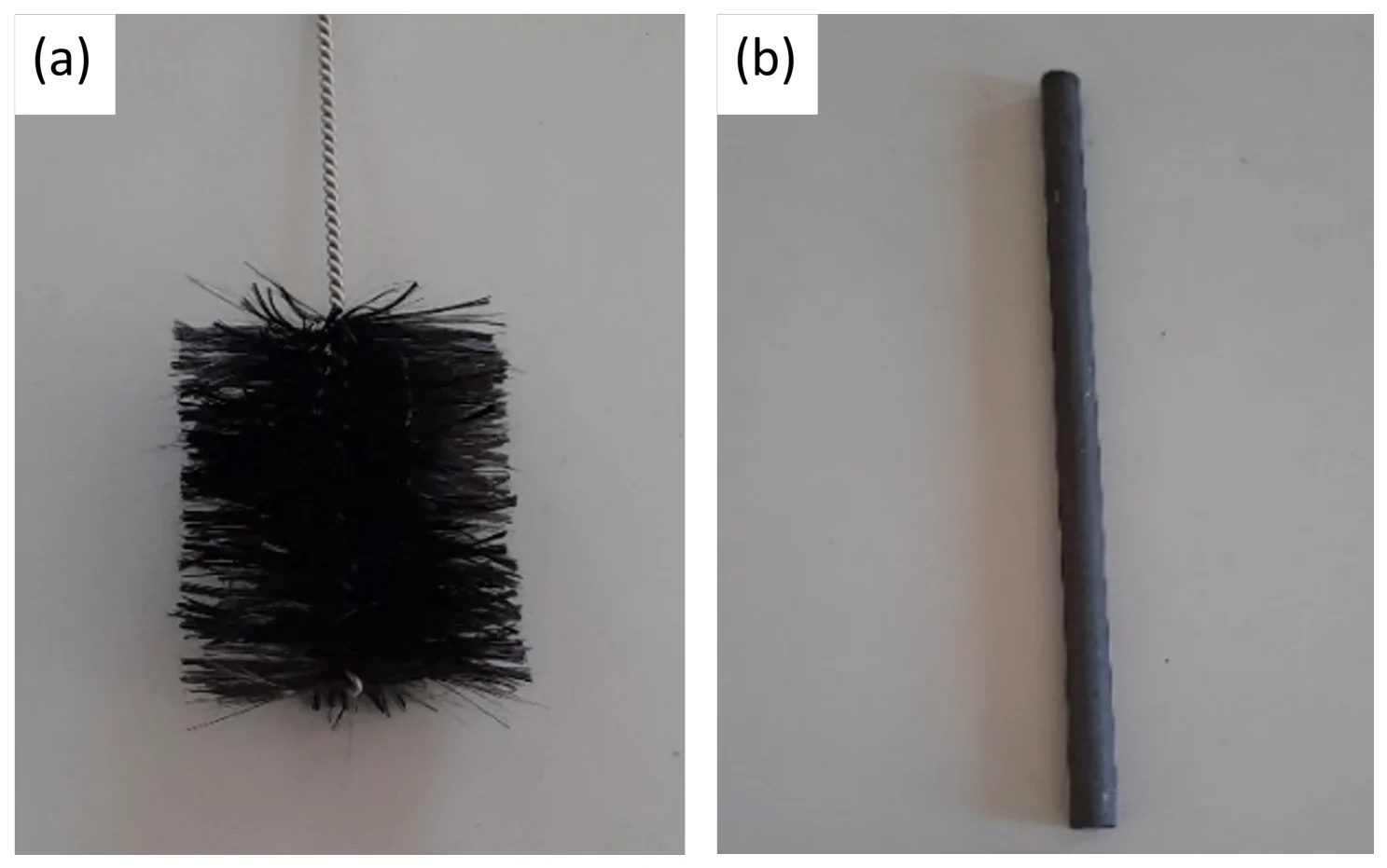
(**a**) Carbon brush as an anode and (**b**) carbon rod as a cathode.

The substrate mixture, prepared on-site on the day the experiment started, was derived from cow dung with a specific gravity of 1.22 and further supplemented with dissolved beetroot molasses [9,19,30,31,32,33]. The cow-manure inoculum was used without pre-acclimation. All five cells were prepared and initiated under the same conditions, and electrical monitoring began immediately after the substrate was introduced and the external circuits were connected. This approach allowed the complete operational development of the cells to be monitored, including initial microbial adaptation and later-stage operation under outdoor conditions. No separate voltage threshold was used to define startup completion, as performance was evaluated over the full 40-day experimental period. The cow manure served both as an organic substrate component and as the source of the mixed microbial inoculum. No selected microbial culture was added, and microbial community characterization was not included in the experimental design [6]. The initial pH of the complete manure–molasses–water substrate was not measured. An available COD value of 27,000 mg/L represented the cow-manure component before preparation of the complete substrate and was therefore not treated as the initial COD concentration of the mixture introduced into the reactors. Consequently, cell-specific COD removal efficiencies were not calculated. Carbon brushes, shown in Figure 3a, specifically designed for wastewater treatment, were used as anodes, while cathodes were 20 cm long carbon rods as shown in Figure 3b. The anode and cathode were connected through a fixed external resistance of 1 kΩ. The same resistance was applied to all five reactors to maintain a consistent electrical loading condition. The value was selected based on previous MFC studies using comparable wastewater and organic substrates. Internal resistance was not measured directly, and polarization curves were not generated. Therefore, the reported power values represent performance under the selected 1 kΩ load rather than confirmed maximum-power-point values.

## 3. Results and Discussion

This section presents the findings of the 40-day experiment on five DCMFCs, showing trends in electricity generation and the influence of variables like mixing, aeration, and solar insolation. Because monitoring began immediately after reactor preparation, the early measurements capture the initial adaptation and development of the cells, while the subsequent measurements reflect their later operational behaviour.

**Figure 4:**
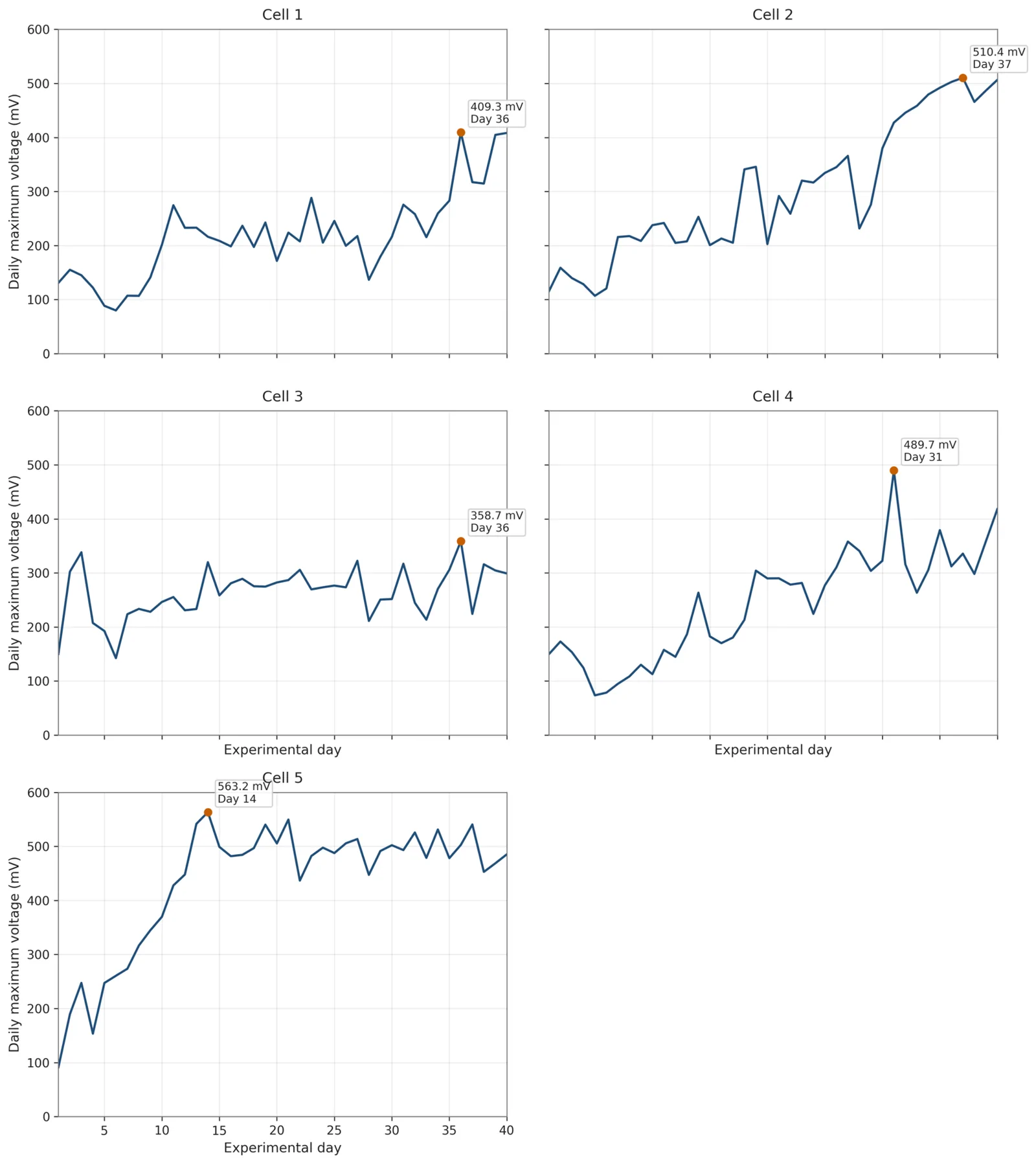
Daily maximum voltage for each cell.

Figure 4 shows the evolution of voltage outputs for each unique configuration (Cells 1, 2, 3, 4, and 5) over the duration of the experiment. The y-axis represents the voltage measurements in millivolts (mV), whereas the x-axis represents the chronological evolution of the experiment, day by day. Cell 5, having mixing and aeration operated for 24 h daily, appears as the top performer, achieving its peak at 563.2 mV on the 14th day. Cell 2, incorporated with controlled aeration for 5 h, reached its highest voltage output of 510.4 mV on day 37, while Cell 4, fitted with controlled aeration and mixing, peaked at 490.3 mV on day 31. Cell 1, the baseline, and Cell 3, controlled mixing, recorded relatively lower voltages, with maximum voltage outputs of 409.3 mV and 358.7 mV, respectively.

Cell 5, operated with continuous mixing and aeration, reached the highest voltage of 563.2 mV on day 14. Cell 2, operated with controlled cathodic aeration for 5 h/day, reached its maximum voltage of 510.4 mV on day 37. Cell 4, operated with controlled aeration and mixing, reached a maximum voltage of 489.7 mV on day 31. The baseline Cell 1 and mixing-only Cell 3 reached maximum voltages of 409.3 and 358.7 mV, respectively. A notable observation is the clustering of peak voltage outputs for most cells (Cells 1, 2, 3, 4) between days 31 and 37. This could suggest optimal environmental or operational conditions conducive to improved microbial activity and energy conversion.

However, Cell 5 showed resilience after its initial voltage surge. Even after reaching its peak on day 14, it steadily maintained a voltage threshold above 463.7 mV. This can be attributed to its unique configuration, continuous operation coupled with an aerator and mixer. This combination promotes aeration and ensures efficient mixing, facilitating rapid and efficient chemical reactions within the cell. The overall increase in voltage over time is consistent with the progressive development of the cells following inoculation, including microbial adaptation and biofilm establishment. The comparison therefore reflects performance across the complete commissioning-to-operation period rather than steady-state operation alone.

**Figure 5:**
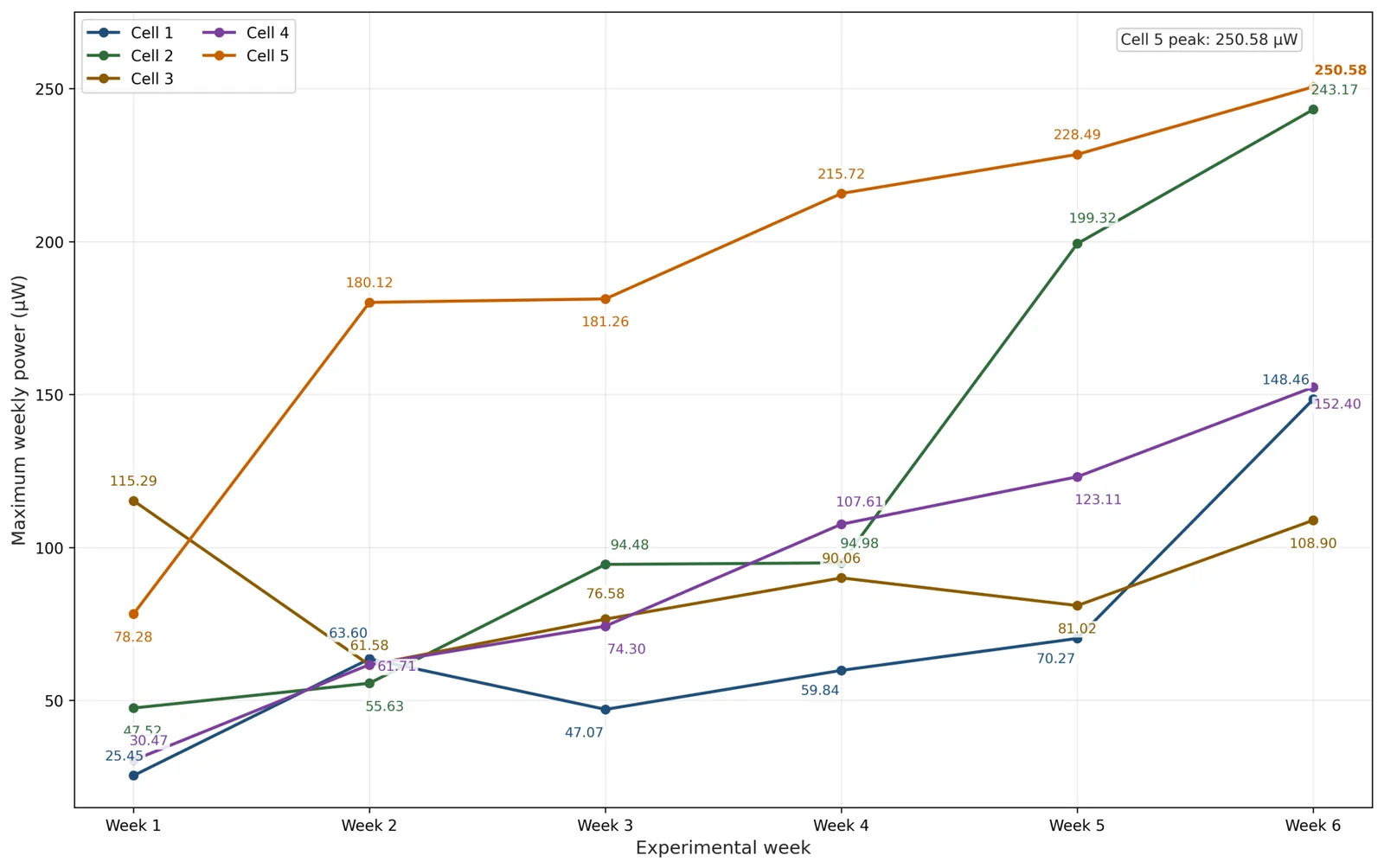
Maximum weekly power generation in µW for each cell.

Figure 5 visualizes the weekly maximum power generation across the five cells throughout the experiment. While each cell demonstrates its unique trajectory and a distinct variation in power generation across the cells, a general upward trend in power generation is noticeable, emphasizing consistent improvements as time progresses. Cell 5 recorded the highest weekly power values among the configurations during most of the experimental period, achieving a maximum power generation of 228.49 µW.

Remarkably, while Cell 4 did not match the power outputs of Cells 5 or 2, its power generation pattern closely resembled the trend of Cell 5. This similarity suggests that the underlying mechanisms or factors affecting power generation in Cells 4 and 5 might have some commonalities.

Figure 5 presents the maximum weekly power recorded for each configuration under the fixed 1 kΩ external load. Cell 5 reached the highest overall power of 250.58 µW in Week 6, followed closely by Cell 2 at 243.17 µW. The overall maximum values for Cells 1, 3, and 4 were 148.46, 115.29, and 152.40 µW, respectively. These results describe gross electrical output under the tested load and should not be interpreted as projected future performance or as maximum-power-point measurements.

### 3.1. The Effect of Aeration and Mixing on the Maximum Power Density

**Figure 6:**
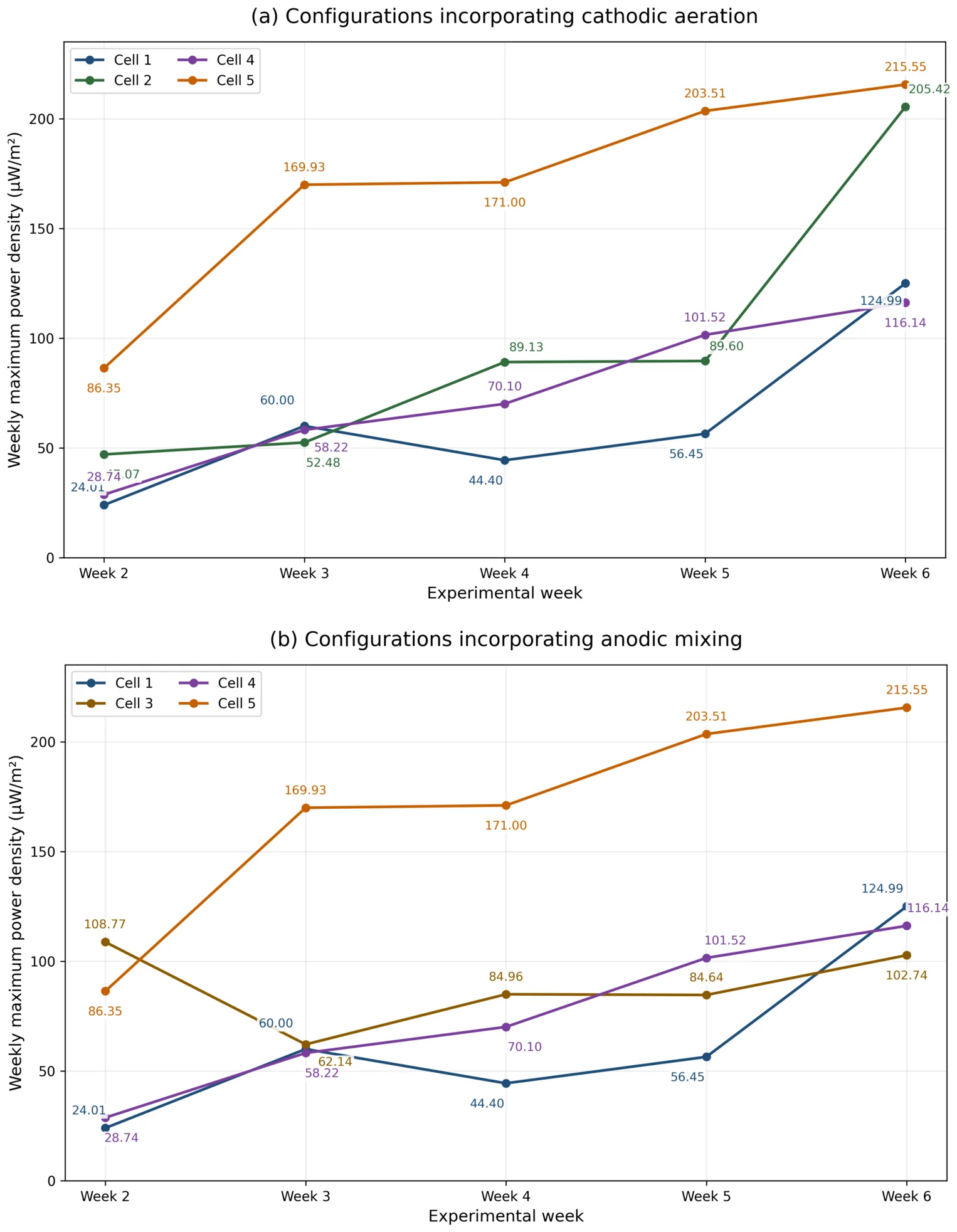
Effect of (**a**) aeration and (**b**) mixing on power density.

The findings from this experimental study emphasize the fundamental role of cathodic aeration in enhancing the power density as shown in Figure 6a. This observation is specifically evident in Cells 2, 4, and 5. The observed response can be interpreted in relation to the different functions of the two active components. Cathodic aeration can increase oxygen availability near the cathode, reduce oxygen mass-transfer limitations, and support the cathodic oxygen-reduction reaction. Anodic mixing can improve substrate distribution, biomass suspension, and mass transport between the bulk substrate and the anode-associated microbial community, thereby increasing substrate accessibility to the biofilm [10,11]. These effects are not necessarily proportional to operating duration because excessive or continuous operation also increases auxiliary energy consumption and may alter local hydrodynamic conditions. The results are therefore interpreted as configuration-specific observations under the tested 5 h and continuous operating schedules rather than as evidence of a universally optimal operating regime. The plots represent the weekly maximum power density values for weeks 2 through 6. Week 2 started on day 2, and week 6 ended on day 36. Weeks 1 and 7 were excluded from the chart because they did not provide enough information for seven consecutive days; however, they followed the same trend.

Table 2 compares the Week 6 maximum power densities of the configurations incorporating cathodic aeration. Power density represents absolute power normalized by the manufacturer-specified anode surface area of 1.06 m^2^. Baseline Cell 1 recorded 124.99 µW/m^2^. Relative to this value, Cell 2 recorded 205.42 µW/m^2^ and was 64.3% higher, Cell 4 recorded 116.14 µW/m^2^ and was 7.1% lower, and Cell 5 recorded 215.55 µW/m^2^ and was 72.5% higher. These percentages are descriptive comparisons under the tested conditions and should not be interpreted as statistically confirmed treatment effects.

As for manure substrate, mixing in the anodic chamber remains a relatively under-researched domain in MFC studies. Figure 6b indicates a consistent surge in power densities for Cells 3, 4, and 5, attributed to manure mixing during the anaerobic digestion. This insight suggests that the integration of manure mixing within the anodic chamber can increase MFC performance metrics, resulting in heightened power densities. Although Cell 3, having a mixer only, showed a slower increasing trend in terms of weekly maximum power density, the overall average power density increase compared to the baseline is 71.07%. Under the tested conditions, Cell 5 recorded the highest Week 6 maximum power density, although the experimental design does not support a conclusion of statistical superiority among the configurations. Although continuous mixing and aeration produced the highest gross electrical output, the auxiliary energy required by the active components must also be considered. The 6 W mixer and 2.2 W aerator together required a continuous input of 8.2 W, equivalent to approximately 196.8 Wh/day and 7872 Wh over the 40-day experimental period. The continuous configuration was therefore not energy self-sufficient. Cell 5 should be interpreted as the highest gross-output configuration under the tested conditions rather than as a net-positive or energy-optimized design. Intermittent, lower-power, or demand-responsive operating schedules should be investigated before practical scale-up.

### 3.2. Effect of Solar Insolation and Temperature on Power Generation

**Figure 7:**
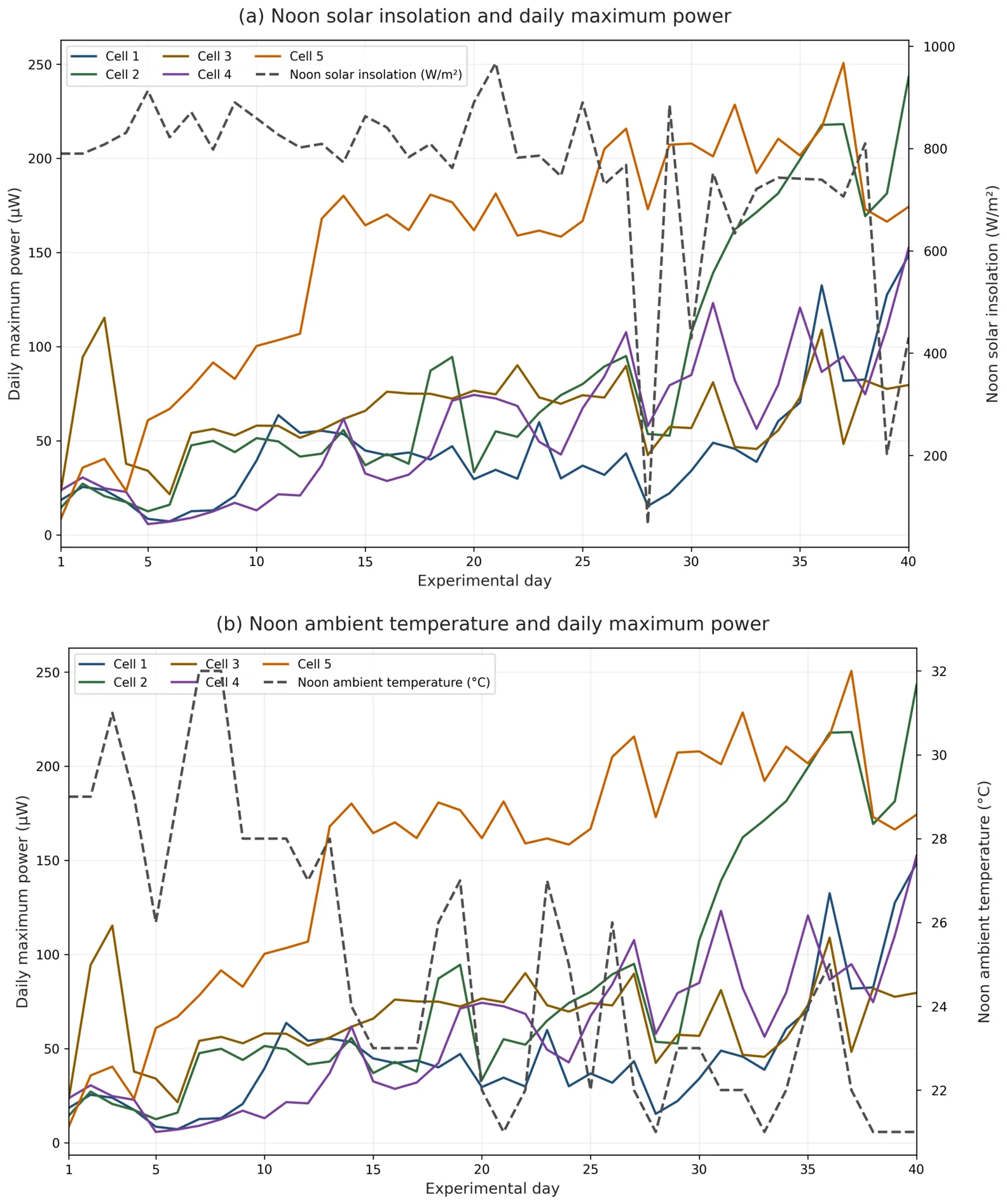
Daily maximum power compared with corresponding noon measurements of (**a**) solar insolation and (**b**) ambient temperature during the 40-day outdoor experiment.

Figure 7 compares daily maximum power with the corresponding noon solar insolation and ambient temperature measurements. Pearson correlation analysis indicated that the relationships between the environmental measurements and daily maximum power differed among the five configurations. Solar insolation was negatively correlated with daily maximum power in Cell 1 (r = −0.381, *p* = 0.015), Cell 2 (r = −0.418, *p* = 0.007), and Cell 4 (r = −0.444, *p* = 0.004). The associations for Cell 3 (r = 0.028, *p* = 0.862) and Cell 5 (r = −0.277, *p* = 0.083) were not statistically distinguishable from zero.

Ambient temperature was negatively correlated with daily maximum power in Cell 1 (r = −0.415, *p* = 0.008), Cell 2 (r = −0.544, *p* < 0.001), Cell 4 (r = −0.710, *p* < 0.001), and Cell 5 (r = −0.757, *p* < 0.001). The association for Cell 3 was weaker and was not statistically distinguishable from zero (r = −0.177, *p* = 0.274).

The observed negative associations may reflect several concurrent factors, including changes in microbial activity, oxygen solubility, mass transport, and the development of the reactors during the experimental period.

These correlations are exploratory and should not be interpreted as direct causal environmental effects because solar insolation, ambient temperature, microbial adaptation, and reactor development changed concurrently over time. Only ambient temperature was monitored; substrate temperature within the individual reactors and the temperature, conductivity, and physical stability of the agarose salt-bridge separator were not measured.

Wind speed was monitored as a contextual outdoor parameter but was not included in the correlation analysis because the measurements represented general ambient conditions rather than reactor-specific airflow. Reactor-level airflow, evaporative loss, and surface-temperature measurements were not available to support a direct interpretation of wind effects on electrical performance. For wastewater treatment, COD quantifies the oxygen needed to convert organic matter in water to inorganic substances. It evaluates biodegradable and non-biodegradable organic content and serves as a robust metric. After 40 days, wastewater samples from the five test cells were analyzed for COD.

Table 3 lists the COD percent change in relation to the baseline design, Cell 1. Under the stringent standards of SM 5220 B, 2011 [34], testing was conducted at the Royal Science Society Laboratories in Amman, Jordan. After 40 days, Cells 2 and Cell 5 had final COD concentrations of 55,600 and 54,400 mg/L, respectively. These concentrations were 10.5% and 12.4% lower than the final concentration in baseline Cell 1. The percentages represent endpoint differences relative to the baseline reactor. Cell 3 and Cell 4 had final COD concentrations 7.1% and 12.7% higher than the baseline, respectively. These endpoint differences may reflect resuspension of settled particulate organic matter caused by mixing, heterogeneity in sampling the high-solid substrate, or concentration effects during outdoor operation. Because only one final COD sample was analysed from each reactor and no intermediate measurements were available, the relative contribution of these mechanisms cannot be determined.

The uncertainties associated with the voltage and current measurements were recorded, aligning with the multimeter’s specifications provided by the manufacturer, CEM, and were rated at ±(1.0%+3) and ±(1.2%+3), respectively. The power uncertainty was determined based on the combined uncertainties of voltage and current using the established law of propagation. Table 4 lists the maximum values and the uncertainties.

The highest recorded value in the experiment was observed for voltage in Cell 5, with a value of 563.2 mV and an uncertainty of ±(5.932), representing the highest degree of uncertainty in this study. The reported uncertainty values fall within the acceptable range of uncertainties stipulated in the relevant literature. Any result with a percentage uncertainty of 10% or less can be considered reliable [35].

Practical implementation requires consideration of more than gross electrical output. Recent scale-up assessments emphasize that MFC deployment depends on reactor stability, membrane and electrode durability, internal resistance, fouling control, material cost, treatment consistency, auxiliary energy requirements, and net energy recovery [36]. In the present experiment, continuous aeration and mixing produced the highest observed electrical output, but both active components required external energy. Cell 5 should therefore be regarded as the highest-performing electrical configuration under the tested conditions rather than as an energy-self-sufficient design. Future investigations should quantify the complete net energy balance and assess intermittent, demand-responsive, or lower-energy mixing and aeration strategies before large-scale implementation is considered.

## 4. Conclusions

This study compared five dual-chamber microbial fuel-cell configurations operated outdoors over 40 days using the same cow-manure–beetroot-molasses substrate, electrodes, agarose salt-bridge separator, and monitoring approach. Cell 5, operated with continuous mixing and aeration, reached the highest recorded voltage of 563.2 mV and the highest power under the fixed external load. Compared with baseline Cell 1, Cell 5 recorded a 72.5% higher Week 6 maximum power density under the tested conditions. Its final COD concentration was 12.4% lower than that of Cell 1; however, this represents an endpoint comparison rather than a reactor-specific COD removal efficiency because equivalent initial COD measurements were unavailable for the complete substrate in each reactor.

Continuous operation produced the highest gross electrical output but was not energy self-sufficient, as the auxiliary energy required for mixing and aeration exceeded the electrical energy recovered. Exploratory correlation analysis did not identify a consistent positive relationship between solar insolation and daily maximum power. Negative associations with ambient temperature were observed for several cells, but these relationships may reflect concurrent reactor development and changing outdoor conditions and should not be interpreted as direct causal effects.

The principal campaign used one reactor for each configuration. Sequential reproducibility checks showed comparable trends and approximately 2% variation in maximum power, but independent parallel reactor replication was not conducted. The comparisons should therefore be interpreted as descriptive observations under the tested conditions. Overall, the study provides application-oriented evidence on the behaviour of aeration and mixing strategies under equivalent outdoor conditions. Future work should incorporate independent parallel reactors, polarization analysis, internal-resistance measurements, separator characterization, intermediate COD sampling, and complete net-energy evaluation before system optimization or scale-up is considered.

## Figures and Tables

**Table 1:** MFC setup—cell configuration.

Cell	Carbon Brush (Anode) and Carbon Rod (Cathode)	Membrane	Mixer	Aerator	Daily Period of Operation
Cell 1	Yes	Yes	No	No	N/A
Cell 2	Yes	Yes	No	Yes	5 h
Cell 3	Yes	Yes	Yes	No	5 h
Cell 4	Yes	Yes	Yes	Yes	5 h
Cell 5	Yes	Yes	Yes	Yes	24 h

**Table 2:** Week 6 maximum power density and percentage difference relative to baseline Cell 1 for configurations incorporating cathodic aeration.

Cell	Max Power Density (µW/m^2^)	Percentage of Increase/Decrease in Comparison with the Baseline
Cell 1—Baseline	124.99	–
Cell 2—Aerator (Controlled)	205.42	+64.3%
Cell 4—Aerator + Mixer (Controlled)	116.14	−7.1%
Cell 5—Aerator + Mixer (Constant operation)	215.55	+72.5%

**Table 3:** Final COD concentrations after 40 days and descriptive differences relative to baseline Cell 1.

Test (COD)	Final COD Concentration (mg/L)	Difference Relative to Cell 1
Cell 1	62,100	Baseline Cell—passive design
Cell 2	55,600	−10.5%
Cell 3	66,500	+7.1%
Cell 4	70,000	+12.7%
Cell 5	54,400	−12.4%

**Table 4:** Maximum voltage, current, and power recorded for the five DCMFC configurations, with associated measurement uncertainties.

	Cell 1	Cell 2	Cell 3	Cell 4	Cell 5
Maximum voltage (mV)	409.3	510.4	358.7	489.7	563.2
Voltage uncertainty value	±4.39	±5.40	±3.89	±5.20	±5.93
Maximum current (µA)	368.3	480.1	340.9	364.5	463.6
Current uncertainty value	±4.72	±6.06	±4.39	±4.67	±5.86
Maximum power (µW)	148.5	186.5	115.3	152.4	250.6
Power uncertainty value	±2.48	±3.07	±1.94	±2.54	±4.12

## Data Availability

Dataset available on request from the authors.

## Abbreviations

The following abbreviations are used in this manuscript:

A	Anode Area
AFC	Alkaline Fuel Cell
BES	Bio-Electrochemical System
COD	Chemical Oxygen Demand
DCMFC	Dual-Chamber Microbial Fuel Cell
FC	Fuel Cell
I	Current
MFC	Microbial Fuel Cell
MCFC	Molten Carbonate Fuel Cell
P	Power
Pd	Power Density
PEM	Proton Exchange Membrane
PEMFC	Proton Exchange Membrane Fuel Cell
SOFC	Solid Oxide Fuel Cell
V	Voltage
